# Immunotherapy for Diffuse Large B-Cell Lymphoma: Current Landscape and Future Directions

**DOI:** 10.3390/cancers13225827

**Published:** 2021-11-20

**Authors:** Dipenkumar Modi, Bindu Potugari, Joseph Uberti

**Affiliations:** 1 Department of Oncology, Barbara Ann Karmanos Cancer Institute, Wayne State University, 4100 John R, HW04HO, Detroit, MI 48201, USA; ubertij@karmanos.org; 2 Department of Hematology and Oncology, St Joseph Mercy Health System, Ann Arbor, MI 48201, USA; Bindu.Potugari@stjoeshealth.org

**Keywords:** diffuse large B-cell lymphoma (DLBCL), autologous stem cell transplant (autoSCT), allogeneic stem cell transplant (alloSCT), CAR T-cell therapy, bispecific T-cell engager antibody, immunotherapy, nivolumab, pembrolizumab, checkpoint inhibitors

## Abstract

**Simple Summary:**

Immunotherapy has played a pivotal role in the management of relapsed DLBCL. Stem cell transplant and CAR T-cell therapy are curative treatment modalities for relapsed disease. Despite this, a subset of patients continues to progress, and their outcomes remain dismal. Newer therapeutic options to optimize outcomes as well as minimize toxicity are warranted.

**Abstract:**

Diffuse large B-cell lymphoma (DLBCL) is a heterogeneous disease. B-cell receptor (BCR) pathway is essential for malignant B-cell growth, survival, and proliferation. Various immune cells, including T-cells and macrophages in the tumor microenvironment (TME) contribute to tumor cell survival and pathogenesis of chemo-resistance. The presence of many targets on the malignant B-cells and in the TME has led to emergence of novel therapeutic agents. Stem cell transplant is the oldest treatment modality leveraging immune system in DLBCL. Subsequently, CD20 targeting monoclonal antibody and chimeric antigen receptor (CAR) T-cell therapy changed the treatment landscape of DLBCL. Recently, multiple novel immunotherapeutic agents have been added in the armamentarium for the management of DLBCL, and many are under development. In this review article, we will review latest updates of immunotherapeutic agents in the management of DLBCL.

## 1. Introduction

Diffuse large B-cell lymphoma (DLBCL) is the most common type of non-Hodgkin’s lymphoma (NHL), representing approximately 30–40% of new cases each year. Using gene expression profiling (GEP), it can be classified into two subtypes according to the cell of origin (COO): germinal center B-cell (GCB) and non-germinal center B-cell (non-GCB) [1]. GCB and non-GCB subtypes account for 40% and 60% of de-novo DLBCL, respectively. Patients with the GCB subtype have better outcomes when compared to those with the non-GCB subtype [1,2,3]. Recently, a number of molecular abnormalities have identified subsets of DLBCL which carry unfavorable prognoses. MYC translocation is reported in 10–15% of DLBCL. In the CORAL (Collaborative Trial in Relapsed Aggressive Lymphoma) study, relapsed 8q24/MYC positive DLBCL had an inferior 4-year progression-free survival (PFS) (18% vs. 42%) and overall survival (OS) (29% vs. 62%), with salvage chemotherapy followed by autologous stem cell transplant (autoSCT), when compared to MYC negative DLBCL [4]. Double or triple hit lymphoma (DHL/THL) is a subtype of DLBCL which is characterized by MYC and BCL2 and/or BCL6 gene rearrangements by FISH, and represents 6–14% of DLBCL cases [5]. It carries the worst prognosis among all DLBCL cases [6,7,8,9]. Even if a DHL is deemed chemo-sensitive to salvage chemotherapy, the 4-year PFS after autoSCT is 28% [10]. Double-expressor lymphomas (DEL: Myc positive ≥40% and Bcl2 + in >50% by IHC) account for 40% of newly diagnosed DLBCL and 50% of relapsed refractory DLBCL. DELs have a better prognosis than DHL, however, this is worse than the “standard” DLBCL [7,11]. Relapsed chemo-sensitive DEL patients have a 4-year PFS of 48% after autoSCT when compared to 58% of “standard” DLBCL [10]. DELs are associated with the non-GCB subtype, while DHL/THL are typically associated with the GCB subtype. In a study involving 893 patients with de-novo DLBCL, 66% of DEL patients were of the ABC subtype, and 39% of non-DEL patients were of the ABC subtype [12]. Additionally, 27% of GCB–DLBCL cases express a 104-gene double-hit signature (DHITsig), and only half of these patients have concurrent MYC and BCL2 rearrangements. Patients with DHITsig experience inferior outcomes after R-CHOP than those without DHITsig (5 year time to progression, 57% and 81%, respectively). Moreover, these patients carry poor outcomes regardless of their MYC/BCL2 rearrangement status [13].

With the increased availability of deep genomic sequencing, novel, molecularly defined subsets of DLBCL are being identified. Schimtz et al. identified four genetic subtypes of DLBCL using exome and transcriptome sequencing: MCD (co-occurrence of MYD88L265P and CD79B mutations), BN2 (BCL6 fusions and NOTCH2 mutations), N1 (NOTCH1 mutations), and EZB (EZH2 mutations and BCL2 translocations). The BN2 and EZB subtypes were associated with favorable survival, while the MCD and N1 subtypes carry inferior outcomes when treated with chemoimmunotherapy [14]. Similarly, Chapuy et al. classified DLBCL into five different subgroups according to the presence of low-frequency alterations, somatic copy number alterations (SCNA), and structural variants (SV). These included low-risk ABC–DLBCLs of extrafollicular/marginal zone origin, (C1), high-risk GCB–DLBCLs with BCL2 SVs and alterations of PTEN and epigenetic enzymes, (C3), low-risk GCB–DLBCLs with distinct alterations in BCR/PI3K and JAK/STAT, as well as BRAF pathway components and multiple histones, (C4), and a COO-independent group of tumors with a biallelic inactivation of TP53, 9p21.3/CDKN2A and associated genomic instability, (C2) [15]. Further refinement of the genetic subtypes of DLBCL was performed, which included the MCD, N1, A53, BN2, ST2, MYC+, and MYC− subsets [16]. Furthermore, another study demonstrated five distinct subtypes of DLBCL using genomic sequencing: MYD88, BCL2, SOCS1/SGK1, TET2/SGK1, and NOTCH2 [17]. Although our understanding of genetic subtypes and their association with clinical features and treatment outcomes is increasing with time, it is not routinely used clinically, and currently remains under investigation.

## 2. CD20 Directed Monoclonal Antibody (mAb)

Rituximab is the first immunotherapy used in the treatment of DLBCL. The addition of rituximab to chemotherapy has been shown to be superior in terms of complete remission, as well as event-free, progression-free, and overall survival when compared to chemotherapy alone in newly diagnosed DLBCL patients [18,19,20,21,22,23]. Obinutuzumab is a type II glycoengineered anti-CD20 mAb, and has a superior antibody-dependent cellular cytotoxicity (ADCC) and phagocytosis than rituximab [24]. A randomized phase III trial, comparing addition of obinutuzumab or rituximab to CHOP chemotherapy in previously untreated advanced DLBCL, did not demonstrate any difference in the progression-free and overall survival [25]. Furthermore, rituximab is typically combined with salvage chemotherapy and other therapeutic regimens for the treatment of relapsed refractory DLBCL [26,27,28,29]. We routinely use rituximab in newly diagnosed and relapsed refractory settings.

## 3. Autologous Stem Cell Transplant

Rituximab plus an anthracycline-based chemo-immunotherapy (R-CHOP) is the standard treatment for newly diagnosed DLBCL, and provides long-term remissions in up to 70% of patients [20,23,30]. Approximately 30% of patients eventually relapse. Salvage chemotherapy followed by autoSCT offers durable remissions in approximately half of patients with relapsed DLBCL. In the pre-rituximab era, the PARMA trial compared autoSCT with consolidative chemotherapy in 215 chemo-sensitive relapsed NHL patients. After two cycles of salvage DHAP chemotherapy, 109 patients with chemo-sensitive disease were randomized either to autoSCT or to four additional cycles of DHAP consolidative chemotherapy. AutoSCT resulted in superior response rates (84% vs. 44%), event-free survival (EFS) (46% vs. 12%), and OS (53% vs. 32%) [31]. In the post-rituximab era, the CORAL and the NCIC-CTG LY.12 study were performed to evaluate the impact of the incorporation of rituximab in the salvage setting. The CORAL study randomized relapsed refractory CD20 positive DLBCL patients to either the R-ICE or R-DHAP salvage regimens, and responding patients proceeded to autoSCT. Response rates were similar after three cycles of R-ICE and R-DHAP (63.5% vs. 62.8%, respectively), and no significant difference for the three-year EFS or OS was noted between R-ICE and R-DHAP treatments [28]. Similarly, the Canadian study NCIC-CTG LY.12 compared GDP to DHAP in transplant eligible patients with relapsed refractory aggressive NHL, and reported no difference in the overall response rates (ORR) (45.2% vs. 44%, respectively), EFS, and OS following autoSCT [26,27].

The CORAL study randomized patients with relapsed DLBCL to rituximab maintenance or observation alone following autoSCT and did not observe any difference in the EFS, PFS, or OS [28]. The BMT CTN 0401 study substituted rituximab with radioimmunotherapy, as a part of the BEAM conditioning regimen, and did not observe an improvement in outcomes [32]. The Center for International Blood and Marrow Transplant Research (CIBMTR) registry study did not observe any difference in OS, PFS, relapse, or non-relapse mortality (NRM) with the addition of rituximab to the BEAM conditioning regimen in 862 relapsed chemo-sensitive DLBCL patients undergoing autoSCT [33].

### Factors Affecting Outcomes of AutoSCT

The age adjusted international prognostic index (IPI) score incorporates clinical variables such as age, serum LDH, ECOG performance status, Ann Arbor stage, and extra-nodal involvement. In the CORAL trial, patients with a high IPI score (2–3) experienced a lower EFS when compared to those with a low IPI score (0–1) (18% vs. 40%) [28]. The second line age-adjusted IPI (sAAIPI) score, consisting of LDH, stage III or IV disease, and performance status, predicts PFS and OS in relapsed refractory DLBCL treated with ICE chemotherapy followed by autoSCT. Three-year PFS and OS were 70% and 74% for low-risk sAAIPI (0 factor), 39% and 49% for intermediate risk sAAIPI (1 factor), and 16% and 18% for high-risk sAAIPI (two to three factors) [34].

The timing of disease relapses affects the post-transplant outcomes. Three-year PFS was significantly lower for patients experiencing an early relapse (<12 months) when compared to late relapse (>12 months) in the CORAL trial (20% vs. 45%) [28]. Primary refractory disease portends poor outcomes with salvage chemotherapy followed by autoSCT [27,35,36]. The SCHOLAR-1 study evaluated the outcomes of refractory DLBCL (progressive or stable disease to first-line or salvage chemotherapy, or relapse within 12 months of autoSCT), and revealed a poor response rate to the next line of therapy (ORR 26% and CR 7%), a median survival of 6.3 months and a one-year survival of 28% [37]. The MSKCC study reviewed the outcomes of primary refractory DLBCL patients who underwent salvage chemotherapy followed by autoSCT. Patients were grouped into primary partial responders (partial response (PR) to initial therapy) or primary progressors (minimal or no response to initial therapy) [38]. The Three-year PFS was 49% in primary partial responders compared to 17% in primary progressors. Moreover, the overall response rate (complete response (CR), PR) was 68% in the primary partial responders versus 40% in the primary progressors. Patients with a Deauville response from one to three had a 3-year PFS of 68% when compared to 30% in Deauville score four patients and 0% in Deauville score five patients [38]. Recently, phase III studies comparing CAR T-cell therapy with the standard of care (autoSCT) in relapsed refractory DLBCL patients have completed accrual and results are awaiting (NCT03391466, NCT03570892, NCT03575351).

The PET scan response after salvage chemotherapy is predictive of the autoSCT outcomes [39]. Patients with a Deauville score of one to three in response to salvage chemotherapy experienced a higher three-year PFS and OS of 77% and 86%, respectively, compared to 49% and 54% [40]. Another study evaluated the impact of pre-transplant PET scans following salvage chemotherapy. PET-negative CR after salvage was associated with an improved four-year PFS (64% vs. 32%) and OS (75% vs. 56%) when compared to PET-positive patients [41]. Patients with PR from salvage chemotherapy are at higher risk of failure than CR [40]. A recent CIBMTR registry analysis showed that patients with a chemo-sensitive PET-positive PR following salvage chemotherapy experienced a five-year PFS of 40% after autoSCT [42]. Based on the above-mentioned evidence, we routinely perform PET scans after salvage chemotherapy. We consider autoSCT in chemo-sensitive relapsed DLBCL patients who have achieved partial or complete remission (Deauville score one to three) to salvage chemotherapy, and reserve CAR T-cell therapy to patients with stable or progressive disease (Deauville score four to five). We do not routinely offer rituximab maintenance therapy after autoSCT.

## 4. Allogeneic Stem Cell Transplant

Allogeneic stem cell transplant (alloSCT) is one of the curative treatment modalities in DLBCL. However, its utility is limited partly due to a high non-relapse mortality (NRM), and the availability of newer therapeutic options, including CAR T-cell therapy. Although CRS and ICANS caused by CAR T-cell therapy can be life threatening, NRM is typically low. Moreover, the responses to CAR T-cell therapy are independent to the disease burden, which makes it an attractive treatment option in chemo-refractory disease when compared to alloSCT. Thus, CAR T-cell therapy has essentially replaced alloSCT as a preferred treatment modality in those patients who have failed multiple lines of therapy, and those with post-autoSCT relapse. A recent CIBMTR analysis demonstrated a remarkable decline in the use of alloSCT for DLBCL and a sharp increase in the use of CAR T-cell therapy. Although CAR T-cell therapy offers durable remissions in relapsed DLBCL patients, approximately 50% of these patients still experience disease relapse, and their outcomes are dismal [43,44]. AlloSCT has a role in such circumstances [45,46]. Moreover, alloSCT has been shown to provide durable remissions in high-risk DLBCL, such as DHL and DEL [47], or in disease that has been unresponsive to multiple prior therapies. AlloSCT can be considered in situations where CAR T-cell therapy is not feasible, such as refractory cytopenia or the presence of myelodysplastic syndrome. Unlike CAR T-cell therapy, which specifically targets the CD19 antigen on lymphoma cells, alloSCT offers a strong immune response against multiple unknown tumor antigens through the graft-versus-lymphoma effect that is exerted by the donor stem cells [48,49].

Table 1 summarizes the outcomes of alloSCT for DLBCL. The CIBMTR study evaluated the outcomes of 503 DLBCL patients who underwent alloSCT after disease progression, following prior autoSCT [46]. The three-year NRM, relapse, PFS, and OS were 30, 38, 31, and 37%, respectively. A Karnofsky performance status (KPS) of <80%, chemoresistance, an autoSCT to alloSCT interval of <1 year, and myeloablative conditioning were associated with an inferior PFS. Similarly, the European Society for Blood and Marrow Transplantation (EBMT) analysis reported the outcomes of alloSCT in 101 DLBCL patients who progressed after autoSCT [50]. At 3 years, the NRM was 28.2%, the relapse rate was 30.1%, the PFS was 41.7%, and the OS was 53.8%. A high NRM was noted in older patients (≥45 years) and in those with an early relapse (<12 months) after autoSCT. The relapse rate was high in refractory patients, and a time interval to relapse after autoSCT of <12 months was associated with a lower PFS. In addition, recent studies have shown a higher rate of NRM and a lower relapse rate with myeloablative conditioning regimens (MAC) when compared to reduced intensity conditioning regimens (RIC) in alloSCT treatment for NHL, offsetting the survival advantage associated with MAC regimens [49,51]. Our practice corroborates with previous studies, and we typically reserve alloSCT for post-CAR T-cell relapse, the presence of concurrent myelodysplasia, or the failure of CAR T-cell therapy production.

## 5. Chimeric Antigen Receptor T-Cell Therapy

**Figure 1:**
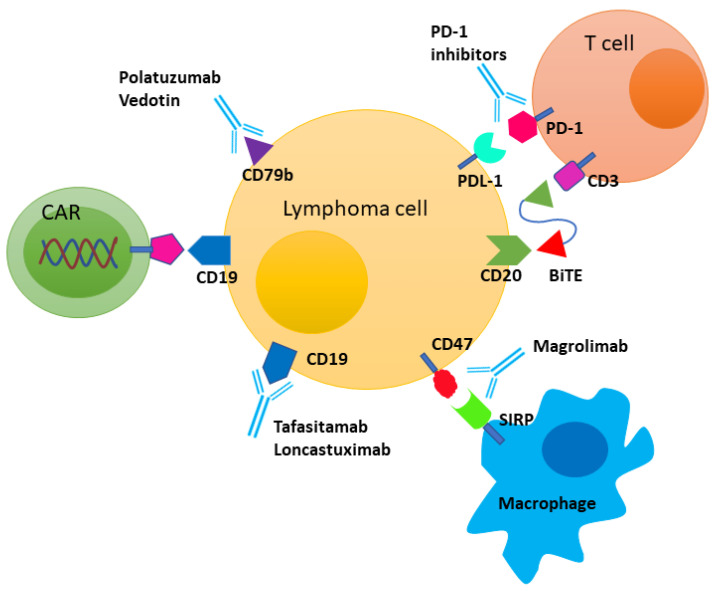
Overview of the interaction between malignant B-cells and other immune cells in the tumor microenvironment, and the therapeutic targets in diffuse large B-cell lymphoma. CAR: Chimeric antigen receptor; BiTE: Bispecific T-cell engager; SIRP: Signal regulatory protein.

The chimeric antigen receptor (CAR) consists of an extracellular antigen recognition domain, which binds to cell surface antigens, and an intracellular signaling domain that provides an activation signal. T-cell receptor activation requires two signals: the first signal is delivered through the T-cell receptor, and the second signal is provided by a costimulatory molecule that allows the proliferation of T-cells. The universal presence of CD19, CD20, and CD22 antigens on malignant B-cells make them the perfect targets for cellular therapies. CD19-directed autologous chimeric antigen receptor T-cell (CAR T-cell) therapy has revolutionized the treatment paradigm for DLBCL (Figure 1). Currently, axicabtagene ciloleucel (axi-cel, Yescarta^®^), tisagenlecleucel (tisa-cel, Kymriah^®^), and lisocabtagene maraleucel (liso-cel, Breyanzi^®^) have been approved by the US Food and Drug Administration (FDA) for the treatment of relapsed refractory DLBCL. The ZUMA-1 multicenter phase II single arm trial evaluated axi-cel in DLBCL, primary mediastinal B-cell lymphoma (PMBCL), and transformed follicular lymphoma (FL), and those which were refractory to prior therapy or had relapsed following autoSCT. The overall response rate (ORR) was 83% and the CR rate was 58%. At a median follow-up of 27.1 months, the median duration of response was 11.1 months, the median OS was not obtained, and the median PFS was 5.9 months [55]. Moreover, one third of the patients with (11/33) PR and half of the patients (11/24) with stable disease at 1 month achieved CR at 6 months. Similarly, the JULIET trial evaluated tisa-cel in relapsed refractory DLBCL patients who were ineligible for autoSCT, or who had disease progression following autoSCT and demonstrated an ORR of 52%, and a CR of 40% [56]. Approximately 54% of patients with PR achieved CR. One-year survival was 49%. Liso-cel is a third CD-19-directed autologous CAR T-cell product with a 4-1BB co-stimulatory domain, which is administered as a sequential infusion of two components (CD8^+^ and CD4^+^ CAR T-cells) at equal target doses (1:1). The TRANSCEND NHL 001 study evaluated liso-cel in relapsed refractory DLBCL, PMBCL, transformed DLBCL from indolent lymphoma, and FL grade 3B. At a median follow-up of 18.8 months, the ORR was 73%, and the CR rate was 53% [57]. Various large, retrospective reports of real-world experiences in using CAR T-cell therapy have shown a similar efficacy [58,59,60,61,62] (Table 2). Given the comparable efficacy and toxicity profiles of approved CAR T-cell therapies, currently the choice of therapy is largely guided by patient’s insurance.

It is important to mention that above-mentioned studies differ in their efficacy and toxicities mainly due to the differences in the study design, population, and lymphodepleting chemotherapy. The ZUMA-1 study reported higher ORR and CR rates when compared to other studies, where bridging chemotherapy was not allowed. It is likely that the ZUMA-1 study may have included patients with a relatively indolent disease biology when compared to others [55,56,57]. Furthermore, the UK study reported a lower ORR when compared to other studies, which could be secondary to the prolonged time from the patient selection to CAR T-cell infusion (median 63 days) [62]. The CD28 costimulatory domain results in rapid T-cell expansion, which leads to an earlier and more severe cytokine release syndrome (CRS), while the use of the 4-1BB costimulatory domain results in a slower expansion of T-cells, as well as a lower incidence and severity of both CRS and immune effector cell-associated neurotoxicity syndrome (ICANS). CAR T-cell therapy is well tolerated in patients with an advanced age [63], multiple comorbidities or a borderline performance status [60], and side effects are, in general, manageable. Several strategies have been employed to mitigate the toxicities that are associated with CAR T-cell therapy. Recently, the use of prophylactic corticosteroids (oral dexamethasone 10 mg daily day zero through day two) has shown to reduce the incidence and severity of CRS (incidence 80%, all grade ≤ 2) and ICANS (incidence 58%, grade 3 ≥ 13%) following axi-cel administration [64]. No impact on the response rate was noted. CAR T-cell therapy is known to induce granulocyte-macrophage colony-stimulating factor (GM-CSF) production, which mediates inflammatory reactions and neurotoxicity. The ZUMA-19 trial, investigating lenzilumab (an anti-GM-CSF monoclonal antibody) in combination with axi-cel in relapsed refractory large B-cell lymphoma, is undergoing [65]. Given the improvement in CRS and ICANS with prophylactic corticosteroids, we routinely offer dexamethasone 10 mg daily for 3 days following axi-cel infusion.

Outcomes of the patients who progress following CAR T-cell therapy are poor [43]. The US lymphoma CAR T-cell consortium reported the outcomes of 136 patients (out of 275, 49%) who had experienced disease progression after axi-cel treatment. The median OS from disease progression was 6 months. Among 74% who received further therapy, the ORR was 29%, with a CR of 17%, and a median PFS of 55 days. CD19 losses were found in approximately 30% of patients at progression [44]. Antigen escape is one of the mechanisms of CAR T-cell resistance, where the malignant cells that are treated with CAR T-cells display the partial or complete loss of the CD19 antigen. To overcome this problem, dual CAR T constructs or tandem CAR T, which is a single CAR construct that contains two single-chain fragment variables (scFvs) to concomitantly target multiple target tumor antigens, have been used [66]. In addition, dual-targeted CAR T-cells (CD19/CD22, CD19/CD20) have shown promising activity in acute lymphoblastic leukemia (ALL), as well as B-cell malignancies [67,68,69]. AUTO3, anti-CD19/CD22 CAR-T treatment, with or without pembrolizumab, has demonstrated an ORR of 64% and a CR of 55% in a phase I/II study of relapsed refractory DLBCL [70]. CAR T-cell exhaustion and immunosuppressive TME are other plausible etiologies of CAR T-cell failure. PD1 blockade with pembrolizumab has demonstrated an ORR of 27% in patients with a progression of NHL after CD19 CAR T-cell therapy [71]. The ZUMA-6 investigated the safety and efficacy of axi-cel in combination with atezolizumab (four doses every 3 weeks) in relapsed refractory DLBCL patients and noted an ORR of 90% with a CR of 60%. CAR T-cell expansion was greater than two-fold higher than observed in the ZUMA-1 trial [72].

A proportion of patients with a proliferative disease could not receive CAR T-cell therapy in a timely manner; this was either due to a manufacturing failure or delays. The median time from enrollment to infusion was 54 days in the JULIET trial [56], and the median time from leukapheresis to axi-cel delivery was 17 days in the ZUMA-1 study [55]. Approximately 31% of enrolled patients in the JULIET trial could not receive CAR T-cell infusion secondary to disease progression, and around 7% had a manufacturing failure. Similarly, approximately 10% of patients in the ZUMA-1 trial did not receive CAR T-cell infusion. Moreover, T-cell dysfunction with a decrease in functional T-cells is more prevalent in patients who have had multiple lines of prior therapy. Allogeneic CAR T-cells (off the shelf CAR T) can overcome these factors, limiting the access of CAR T-cell therapy to high-risk populations. The off-the-shelf, allogeneic CAR T-cell product, PBCAR0191, has shown a CR of 33% (standard lymphodepletion) and 71% (escalated lymphodepletion) in 13 patients with CD19-positive relapsed refractory NHL. Neither GVHD nor the presence of a CRS or ICANS grade of over three was noted [73]. Table 3 shows the selected ongoing studies of allogeneic and dual-construct CAR T-cell therapy.

## 6. Immune Checkpoint Inhibitor Therapy

The programmed death-1 (PD-1) receptor is expressed by activated T-cells, B-cells, NK cells, and macrophages. It regulates the T-cell-mediated immune response through binding to its ligands, programmed cell-death protein-1 ligand-1 (PD-L1) and PD-L2. Tumor cells express PD-L1 and PD-L2, which subsequently down-regulates T-cell activation, and indirectly helps them to escape the immune response. Upregulation of CTLA-4 in T-cells is another mechanism through which tumor cells suppress the immune response. (Figure 1). In Hodgkin’s Lymphoma (HL), an amplification of 9p24.1 has been shown to increase the expression of PD-L1 and PD-L2, and is associated with a shorter PFS [74]. Subsequently, PD-1 blockade by the anti-PD-1 antibodies nivolumab and pembrolizumab has shown promising results in relapsed [75,76,77,78] and newly diagnosed [79,80] HL.

Unlike HL, DLBCL cells do not frequently express PD-L1. PD-L1 gene alteration has been reported in approximately 25–31% of DLBCL patients [81,82]. A study evaluated the impact of PD-L1 positivity on tumor cells and their microenvironment (mPD-L1) in DLBCL. The expression of PD-L1 on tumor cells and mPD-L1 was observed in 11% and 15.3% of cases, respectively. PD-L1 and mPD-L1-positive DLBCL were significantly associated with the non-GCB subtype and Epstein-Barr virus positivity. Patients with PD-L1-positive DLBCL had a poor OS when compared to PD-L1-negative DLBCL patients, while no difference in OS was observed between mPD-L1-positive and mPD-L1-negative DLBCL patients [83]. Likewise, another study reported that DLBCL with PD-L1 alterations experienced an inferior PFS following front-line chemoimmunotherapy; however, the in relapsed setting, PD-L1 alterations were associated with a response to anti-PD-1 antibodies [82].

Checkpoint inhibitors (CPI) have shown disappointing results in DLBCL. Nivolumab was evaluated in relapsed refractory lymphoma and multiple myeloma in a phase I, multicenter study. Among eleven patients with DLBCL, the ORR was 36% (CR = 18% and PR = 18%), and the median PFS was only 7 weeks. At a median follow-up of 22.7 weeks for DLBCL patients, one of the four patients had a continued response [84]. Checkmate 036, the combination of nivolumab plus ipilimumab, yielded an ORR of 20% in relapsed refractory NHL patients (10/15 with DLBCL), with a median PFS of only 1.5 months [85]. Pidilizumab, an-anti-PD-1 monoclonal antibody, was evaluated in a phase II study of DLBCL patients undergoing autoSCT. In patients without progressive disease, pidilizumab was offered at every 42 days for 3 cycles, beginning 30 to 90 days after autoSCT. The ORR among patients with a measurable disease post-autoSCT was 51%, and the CR and PR rates were 34% and 17%, respectively. The 16 month PFS from the first treatment was 72%. Among those patients who had remained PET-positive at the conclusion of salvage therapy, the 16 month PFS was 70% [86]. Furthermore, pembrolizumab treatment, as a maintenance therapy in chemo-sensitive DLBCL patients undergoing autoSCT, revealed an 18 month PFS of 59% [87]. Recently, a phase II study evaluating the effect of treatment with nivolumab, 3 mg/kg every 2 weeks, in relapsed refractory DLBCL patients who were ineligible for autoSCT, or had failed autoSCT, was conducted. At a median follow-up of 9 months in the autoSCT-failed cohort and 6 months in the autoSCT-ineligible cohort, the ORR values were 10% and 3%, respectively. The median PFS and OS were 1.9 and 12.2 months in the autoSCT-failed cohort, and 1.4 and 5.8 months in the autoSCT-ineligible cohort, respectively. Of the evaluable samples in the analysis of 9p24.1, 16% had a low-level copy gain and 3% contained amplifications. The low response rates in DLBCL were attributed to infrequent genetic alterations in 9p24.1 [88]. The combination of pembrolizumab and R-CHOP was evaluated in 30 newly diagnosed DLBCL cases, and the ORR and CR were 90% and 77%, respectively. At a median follow-up of 25.5 months, the two-year PFS was 83% [89].

Atezolizumab, a humanized IgG1 anti-PD-L1 antibody, in combination with 6 cycles of R-CHOP followed by 12 months of consolidation, was evaluated in 42 untreated advanced DLBCL patients. The ORR was 87.5%, and the two-year PFS and OS were 74.9% and 86.4%, respectively [90]. A number of early clinical trials have shown a modest activity of atezolizumab in combination with various therapeutic agents in relapsed refractory DLBCL patients [91,92]. Durvalumab, a humanized IgG1 anti-PD-L1 antibody, in combination with R-CHOP, has demonstrated a CR rate of 54% in treatment-naïve DLBCL patients [93], and has shown a fair activity in combination with ibrutinib in relapsed refractory DLBCL patients [94]. Ipilimumab, an anti-CTLA-4 antibody, has shown insignificant activity in relapsed refractory B-cell NHL [95,96,97]. Altogether, these data suggest that immune CPI therapies have failed to yield a clinically significant efficacy in DLBCL patients, while the addition of immune CPI therapies to first-line chemoimmunotherapeutic regimens has demonstrated an improvement in responses when compared to historical control rates. However, the long-term survival data are immature, and larger randomized trials are warranted. Ongoing studies of CPI with CAR-T and other agents are shown in Table 3.

## 7. Anti-CD47 Antibody

CD47 or “Do Not Eat Me” is an antiphagocytic signal that is expressed by cancer cells and enables immune evasion by macrophages. Its expression is associated with a poor prognosis. Anti-CD47 antibodies can induce the phagocytosis of tumor cells by the blockade of CD47 and its ligand, SIRPα. Anti-CD47 antibodies induce an antitumor T-cell response. Hu5F9-G4, an IgG4 humanized anti-CD47 monoclonal antibody (mAb), is associated with an ORR of 40% and a CR of 33% in relapsed refractory DLBCL patients [98]. Given the encouraging activity of anti-CD47 antibodies, various trials that incorporate this agent in combination with other regimens are ongoing.

## 8. Bispecific T-Cell Engager (BiTE) Antibody

BiTE antibodies are a newer form of immunotherapy for B-cell NHL. BiTE antibodies target antigens on tumor cells, while another end targets T-cells. It binds to tumor antigen and T-cells simultaneously, and facilitates the T-cell killing of cancer cells (Figure 1). The antigen-experienced T-cell subsets drive cancer cell death, while naïve T-cells are not activated. BiTE antibodies also increase the secretion of cytokines, which lead to changes in the tumor microenvironment. Blinatumomab was the first CD3/CD19 BiTE evaluated in a phase I study of relapsed refractory NHL in 76 patients, and 14 DLBCL patients were included. Among 35 patients who were treated with the maximum tolerated dose of 60 µg/m^2^/day, the ORR was 69%, and 55% for DLBCL patients (CR 36%) [99]. In a phase II study of blinatumomab in relapsed refractory DLBCL patients, the ORR after one cycle was 43%, the CR rate was 19%, and the median PFS was 3.7 months [100]. Blinatumomab was evaluated as a second salvage therapy, following platinum-based first salvage chemotherapy for relapsed refractory NHL, and the ORR at 12 weeks was 37% with a CR rate of 22% [101]. Based on these encouraging results, research into the combination of blinatumomab with lenalidomide (NCT02568553) or pembrolizumab (NCT03340766) is ongoing.

Several next generation BiTE antibodies are under development (Table 4). Notably, these antibodies have shown encouraging activity in relapsed refractory NHL patients, including those who fail CAR T-cell therapy [102,103,104,105]. Moreover, they are available off-the-shelf, and have a prolonged half-life, which allows a more convenient, once-weekly dose. Glofitamab is a BiTE antibody with a 2:1 configuration, allowing for the bivalent binding to CD20 on B-cells, and monovalent binding to CD3 on T-cells [105]. BiTE antibodies have shown encouraging activity and have offered complete remission in patients who have received multiple prior lines of therapy, including CAR T-cell therapy. Currently BiTE antibodies are not FDA approved, and therefore their use remains under investigation. Several trials incorporating these agents in first-line or in salvage settings are ongoing (Table 3).

## 9. Antibody Drug Conjugates (ADC)

Polatuzumab Vedotin (PoV) is an anti-CD79b-directed ADC, and delivers monomethyl auristatin E (MMAE) inside malignant B-cells. The combination of PoV with bendamustine and rituximab (BR) has been shown to improve the CR (40% vs. 17.5%), PFS (median, 9.5 vs. 3.7 months), and OS (median, 12.4 vs. 4.7 months) when compared to BR in transplant-ineligible relapsed refractory DLBCL patients [29]. PoV in combination with lenalidomide and rituximab was evaluated in transplant-ineligible or failed relapsed refractory DLBCL patients. At a median follow-up of 9.5 months, the ORR and CR were 39% and 27%, respectively, and the median PFS and OS were 6.3 and 10.9 months, respectively [106]. Most recently, a phase III study (POLARIX), comparing PoV plus R-CHOP with R-CHOP alone in newly diagnosed DLBCL patients, has finished accrual and results are awaiting.

Loncastuximab tesirine (ADCT-402) is a humanized anti-CD19-directed ADC, delivering a pyrrolobenzodiazepine dimer cytotoxin, SG3199. It has shown an ORR of 48.3%, a CR of 24.1%, and a median PFS of 4.9 months in a phase II trial (LOTIS-2) involving relapse refractory DLBCL patients [107]. Currently, loncastuximab tesirine is being evaluated in combination with R-CHOP in untreated DLBCL patients.

Tafasitamab (MOR208) is an Fc-modified humanized anti-CD19 mAb. In a phase II trial (L-MIND) of relapsed DLBCL patients who were ineligible for transplant, the combination of tafasitamab and lenalidomide demonstrated an ORR of 57.5% and a CR of 40%. At a median follow-up of ≥35 months, the median PFS was 11.6 months, and the median OS was 33.5 months. The median PFS, duration of response, and OS were significantly longer in patients receiving tafasitamab and lenalidomide as second-line therapy when compared to those receiving third- or later-line treatment (23.5 months, 43.9 months, and 45.7 months when compared to 7.6 months, not reached, and 15.5 months, respectively) [108]. A phase II study of tafasitamab in combination with bendamustine when compared to BR in RR DLBCL is ongoing.

All the above-mentioned therapeutic options are non-curative and are often used in relapsed refractory DLBCL to bridge to stem cell transplant or CAR T-cell therapy. Given the impact of BR plus PoV on lymphocyte recovery and peripheral T-cell collection, we typically use this regimen following T-cell collection. Moreover, CD19 expression is typically maintained after the cessation of tafasitamab or loncastuximab tesirine treatments, and prior treatment with either of these agents does not preclude CAR T-cell therapy [109,110]. A study involving 14 DLBCL patients with their disease progressing after loncastuximab tesirine and subsequently undergoing CD19-directed CAR T-cell therapy revealed an ORR of 50% (CR 43% and PR 7%) [110]. Thus, we often consider CD19-directed agents in chemo-refractory patients, to control their disease prior to CAR T-cell therapy.

## 10. Conclusions

Tremendous progress has been made in understanding the molecular pathways that are involved in the pathogenesis of DLBCL. Immunotherapy approaches for the treatment of DLBCL have transformed the therapeutic landscape of the relapsed disease state. Beginning with traditional immunotherapy approaches, such as rituximab and stem cell transplant, the current treatment for DLBCL includes CART-cell therapy and immune checkpoint inhibitors for a subset of patients. Despite the groundbreaking advances in this area, there remain a number of challenges, including the emergence of resistance and toxicity. A multitude of clinical trials utilizing novel treatment strategies to overcome these challenges are underway, with bispecific antibodies being at the forefront. As these new approaches to leverage the host immune response unfold, the next decade is likely to bring a revolution to this area.

## Figures and Tables

**Table 1:** Outcomes of allogeneic stem cell transplants in DLBCL.

Study	No of Pts	Conditioning Regimen	Acute GVHD (Grade 2–4)	Chronic GVHD (1-yr)	NRM (1-yr)	Relapse	PFS	OS
Thomson et al. [52], 2009	48	RIC 100%	17%	22%	29%	33% (4-yr)	48% (4-yr)	47% (4-yr)
Sirvent et al. [53], 2010	68	RIC 100%	39%	41%	23%	41% (2-yr)	44% (2-yr)	49% (2-yr)
Lazarus et al. [54], 2010	79	MAC 100%	42% (100-day)	23%	41%	30% (1-yr)	29% (1-yr)	33% (1-yr)
Van Kampen et al. [50], 2011	101	MAC (37%) vs. RIC (63%)	33%	42%	24.5%	24% (1-yr)	51.5% (1-yr)	64.7% (1-yr)
Bacher et al. [51], 2012	396	MAC (*n* = 165) vs. RIC (*n* = 143) vs. NMA (*n* = 88)	43% vs. 43% vs. 44%	35% vs. 39% vs. 33%	47% vs. 31% vs. 29%	23% vs. 32% vs. 37% (1-yr)	30% vs. 37% vs. 34% (1-yr)	38% vs. 46% vs. 45% (1-yr)
Fenske et al. [46], 2016	503	MAC (25%) vs. RIC (75%)	36%	40%	23%	33% (1-yr)	44% (1-yr)	54% (1-yr)
Modi et al. [49], 2020	70	MAC (67%) vs. RIC (23%)	36.2% vs. 8.7% (Grade 3–4)	27.7% vs. 43.5% (Extensive)	39.7% vs. 39.1% (3-yr)	25.5% vs. 17.4% (3-yr)	34.2% vs. 34.7% (3-yr)	34.4% vs. 43.4% (3-yr)

RIC: Reduced intensity conditioning regimen; MAC: Myeloablative conditioning rmyeegimen; NMA: Non-myeloablative; NRM: Non-relapsed mortality; PFS: Progression-free survival; OS: Overall survival; Yr: Year; GVHD: Graft-versus-host-disease.

**Table 2:** Clinical trials of CAR T-cell therapy in lymphoma.

Trial	CAR-T Product	Costimulatory Domain	No of Pts	Bridging Chemotherapy	Lymphodepleting Chemotherapy	Patient Population	ORR/CR (%)	CRS/ Grade ≥ 3 (%)	ICANS/ Grade ≥ 3 (%)
ZUMA-1 [55]	Axi-cel	CD28	111	No	Fludarabine (Flu) 30 mg/m^2^ and Cyclophosphamide (Cy) 500 mg/m^2^ × 3 days	DLBCL ≥ 2 lines, transformed FL (tFL), PMBCL	82/58	93/13	64/28
JULIET [56]	Tisa-cel	4-1BB	165	Yes (92%)	Flu 25 mg/m^2^ and Cy 250 mg/m^2^ × 3 days, or bendamustine 90 mg/m^2^ × 2 days	DLBCL ≥ 2 lines, tFL	52/40	58/23	21/12
TRANSCEND-NHL 001 [57]	Liso-cel	4-1BB	342	Yes (59%)	Flu 30 mg/m^2^ and Cy 300 mg/m^2^ × 3 days	DLBCL ≥ 2 lines, tFL, PMBCL	73/53	42/2	30/10
Nastoupil et al. [60]	Axi-cel	CD28	165	Yes (53%)	Flu 30 mg/m^2^ and Cy 500 mg/m^2^ × 3 days	DLBCL, PMBCL, tFL	82/64	92/7	69/31
Jacobson et al. [58]	Axi-cel	CD28	122	Yes (45%)	Not available	DLBCL, PMBCL, tFL, transformed marginal zone lymphoma (tMZL), transformed CLL	70/50	93/16	70/35
Sesques et al. [62]	Axi-cel/ Tisa-cel	CD28/4-1BB	70	Yes (97%)	Bendamustine (2%); Flu and Cy	DLBCL, PMBCL, tFL, tMZL	63/48	85/8	28/10
Jaglowski et al. (CIBMTR) [59]	Tisa-cel	4-1BB	70	Not available	Not available	DLBCL, tFL	59.6/38.3	NA/4.3	NA/4.3
Pasquini et al. (CIBMTR) [61]	Axi-cel	CD28	453	Not available	Not available	Large B-cell lymphoma	70/52	83/14	61/NA

ORR: Overall response rate; CR: Complete response; CRS: Cytokine release syndrome; ICANS: Immune effector cell-associated neurotoxicity syndrome; NA: Not available; DLBCL: Diffuse large B-cell lymphoma; FL: Follicular lymphoma; PMBCL: Primary mediastinal B-cell lymphoma.

**Table 3:** Selected ongoing trials of Immunotherapy in DLBCL.

Intervention	Molecular Target	Trial	Phase	Indication	Primary Endpoint	Status
R-CHOP/PoV-R-CHP plus Glofitamab	BiTE (CD3/CD20)	NCT04914741/ COALITION	I/II	Untreated DLBCL	Safety	Recruiting
Glofitamab plus R-CHOP	BiTE (CD3/CD20)	NCT04980222	II	Untreated DLBCL	CR	Recruiting
Mosunetuzumab or in combination with PoV	BiTE (CD3/CD20)	NCT03677154	I/II	Untreated DLBCL	Safety, ORR, CR	Recruiting
Tafasitamab plus lenalidomide plus R-CHOP vs. R-CHOP	Anti-CD19 mAb	NCT04824092/ FrontMIND	III	Untreated DLBCL	PFS	Recruiting
Venetoclax plus PoV plus R-CHP	Anti-CD79b ADC	NCT04790903	I	Untreated DLBCL	Safety	Recruiting
Induction and Maintenance Avelumab	Anti-PD-L1 antibody	NCT03244176	I	Untreated DLBCL	Safety	Recruiting
**Relapsed Refractory Setting**						
Glofitamab plus GemOx vs. R-GemOx	BiTE (CD3/CD20)	NCT04408638	III	RR DLBCL	OS	Recruiting
Epcoritamab vs. standard of care	BiTE (CD3/CD20)	NCT04628494	III	RR DLBCL	OS	Recruiting
Blinatumomab plus lenalidomide	BiTE (CD3/CD20)	NCT02568553	I	RR NHL	Safety	Recruiting
TNB-486 (CD19/CD3 BiTE)	BiTE (CD3/CD20)	NCT04594642	I	RR NHL	Safety	Recruiting
Loncastutixmab plus rituximab vs. R-GemOx	Anti-CD19 ADC	NCT04384484/ LOTIS-5	III	RR DLBCL	PFS	Recruiting
Loncastuximab Tesirine and Ibrutinib	Anti-CD19 ADC	NCT03684694/ LOTIS-3	I/II	RR DLBCL, MCL	Safety	Recruiting
PoV plus R-GemOx vs. R-GemOx	Anti-CD79b ADC	NCT04182204/ POLARGO	III	RR DLBCL	OS	Recruiting
PoV plus R-ICE vs. R-ICE	Anti-CD79b ADC	NCT04833114	III	RR DLBCL	EFS	Recruiting
Nivolumab plus ipilimumab and adaptive T-cell therapy	Anti-PD-1 and CTLA-4 antibody	NCT03305445	Ib/II	RR DLBCL	Safety, CR	Recruiting
Copanlisib and nivolumab	Anti-PD-1 antibody	NCT03484819	II	RR DLBCL, PMBCL	ORR	Recruiting
Pembrolizumab plus anti-CD20 antibody	Anti-PD-1 antibody	NCT03401853	II	RR DLBCL, FL	ORR	Recruiting
Atzolizumab plus R-GemOx	Anti-PD-L1 antibody	NCT03321643	I	RR DLBCL	Safety	Recruiting
Pembrolizumab Plus Vorinostat	Anti-PD-1 antibody	NCT03150329	I	RR DLBCL, FL, HL	Safety	Recruiting
Camrelizumab plus Apatinib	Anti-PD-1 antibody	NCT04476459	I/II	RR DLBCL	ORR	Recruiting
Tislelizumab plus Lenalidomide	Anti-PD-1 antibody	NCT04796857	I/II	RR DLBCL	ORR	Recruiting
Varlilumab plus Nivolumab	Anti-CD27/anti-PD-1antibody	NCT03038672	II	RR NHL	ORR	Recruiting
Nivolumab plus lenalidomide	Anti-PD-1 antibody	NCT03015896	I/II	RR NHL, HL	Safety	Recruiting
DPX-Survivac along or with pembrolizumab with or without low-dose cyclophosphamide	Anti-PD-1 antibody	NCT04920617	II	RR DLBCL	ORR	Recruiting
AUTO3 (CD19/CD22 CAR T) with Pembrolizumab	CAR T/Anti-PD-1 antibody	NCT03287817/ ALEXANDER	I/II	RR DLBCL	Safety, ORR	Recruiting
CD19 CAR-T Expressing IL7 and CCL19 Combined with PD1 mAb	CAR T/Anti-PD-1 antibody	NCT04381741	I	RR DLBCL	ORR	Recruiting
C-CAR066 (anti-CD20 CAR T-cell therapy)	CAR T-cell	NCT04316624	I	RR DLBCL who failed CD19 CAR T-cell therapy	Safety	Recruiting
anti-CD19 and anti-CD20 dual specific CAR T-Cells	CAR T-cell	NCT04486872	I	RR DLBCL	Safety	Recruiting
MB-CART2019.1 (CD19/CD20 CAR T) vs. SOC	CAR T-cell	NCT04844866	II	RR DLBCL	PFS	Recruiting
LUCAR-20S (Anti-CD20 CAR T)	CAR T-cell	NCT04176913	I	RR DLBCL, FL, MCL, CLL	Safety	Recruiting
Autologous Anti-CD20 CAR-T	CAR T-cell	NCT03277729	I/II	RR NHL	Safety	Recruiting
Autologous Anti-CD22 CAR-T	CAR T-cell	NCT04088890	I/Ib	RR NHL	Safety	Recruiting
Autologous anti-CD19/CD20 CAR T	CAR T-cell	NCT04215016	I	RR DLBCL	Safety	Recruiting
Autologous anti-CD19/CD20 CAR T	CAR T-cell	NCT04007029	I	RR NHL, CLL	Safety	Recruiting
Autologous anti-CD19/CD22 CAR T	CAR T-cell	NCT03233854	I	RR NHL	Safety	Recruiting
Acalabrutinib with Anti-CD19 CAR-T	CAR T-cell	NCT04257578	I/II	RR NHL	Safety	Recruiting

PoV: Polatuzumab Vedotin; DLBCL: Diffuse large B-cell lymphoma; RR: Relapsed Refractory; NHL: Non-Hodgkin’s lymphoma; MCL: Mantle cell lymphoma; PMBCL: Primary Mediastinal B-cell lymphoma; FL: Follicular lymphoma; HL: Hodgkin’s lymphoma; CLL: Chronic lymphocytic leukemia; OS: Overall survival; EFS: Event-free survival; ORR: Overall response rate; CR: Complete remission; PFS: Progression-free survival; BiTE: Bispecific T-cell Engager; mAb: Monoclonal antibody; ADC: Antibody drug conjugate.

**Table 4:** Trials of BiTE antibodies in B-cell non-Hodgkin’s lymphoma.

BiTE Antibody	No of Pts	Pt Population	Dosing	ORR (%)		CR (%)		Median PFS (Months)	CRS	ICANS
Epcoritamab [104]	68	R/R NHL (DLBCL = 46; FL = 12; MCL = 4)	SC: 0.0128–60 mg R2PD = 48 mg	DLBCL 12–60 mg = 68%; 48–60 mg = 91%	FL 12–48 mg = 80%	DLBCL 12–60 mg = 46%; 48–60 mg = 55%	FL 12–48 mg = 60%	≥12 mg = 9.1; ≥48 mg = NR	Grade 1–2 = 58%	Grade 3 = 3%
Mosunetuzumab + Polatuzumab [103]	22	R/R NHL (DLBCL = 12; FL3B = 3; tFL = 4; FL 1-3A = 3)	IV: 1-2-60 mg; PoV 1.8 mg/kg every 3 weeks	Aggressive NHL = 63.2%; Post-CAR T = 57.1%	FL = 100%	Aggressive NHL = 47.4%; Post-CAR T = 28.6%	FL = 100%		Grade 1 = 9.1%	None
Odronextamab (REGN1979) [102]	136	R/R NHL (DLBCL = 78)	0.03–320 mg weekly × 12, then every 2 weeks	DLBCL ≥ 80 mg, no Car-T = 55%	DLBCL≥80 mg, prior Car-T = 33%	DLBCL≥80 mg, no Car-T = 55%	DLBCL ≥ 80 mg, Prior Car-T = 21%		61% all grades; Grade 3 ≥ 7%	Grade 3 = 3.7%
Glofitamab [105]	171	R/R NHL (DLBCL = 73; FL1-3A = 44; tFL = 29; Richter’s = 10; PMBCL = 3)	R2PD = 2.5/10/30	Aggressive NHL = 48%; DLBCL = 41.4%	DLBCL ≥ 10 mg = 55.3%; tFL ≥ 10 mg = 64.3%	Aggressive NHL = 33.1%; DLBCL 28.8%	DLBCL ≥ 10 mg = 42.1%; tFL ≥ 10 mg = 64.3%		50.3% all grades; grade 3–4 = 3.5%	43.3%

NHL: Non-Hodgkin’s Lymphoma; R/R: Relapsed refractory; DLBCL: Diffuse large B-cell lymphoma; FL: Follicular lymphoma; MCL: Mantle cell lymphoma; ORR: Overall response rate; CR: Complete remission; CRS: Cytokine release syndrome; ICANS: Immune effector cell-associated neurotoxicity syndrome; R2PD: Recommended phase 2 dose.
